# Influence of TiO_2_ Nanoparticles on the Resistance of Cementitious Composite Materials to the Action of Bacteria

**DOI:** 10.3390/ma14051074

**Published:** 2021-02-25

**Authors:** Andreea Hegyi, Adrian-Victor Lăzărescu, Henriette Szilagyi, Elvira Grebenişan, Jana Goia, Andreea Mircea

**Affiliations:** 1 NIRD URBAN-INCERC Cluj-Napoca Branch, 117 Calea Florești, 400524 Cluj-Napoca, Romania; andreea.hegyi@incerc-cluj.ro (A.H.); henriette.szilagyi@incerc-cluj.ro (H.S.); elvira.grebenisan@incerc-cluj.ro (E.G.); 2 Municipal Hospital, 14–16 1 Mai Street, 405200 Dej, Romania; janagoia@yahoo.com; 3 Facultatea de Construcţii, Technical University of Cluj-Napoca, 28 Memorandumului, 400114 Cluj-Napoca, Romania

**Keywords:** cementitious composites, TiO_2_ nanoparticles, photocatalysis, bactericidal effect

## Abstract

The formation of biofilms on cementitious building surfaces can cause visible discoloration and premature deterioration, and it can also represent a potential health threat to building occupants. The use of embedded biofilm-resistant photoactivated TiO_2_ nanoparticles at low concentrations in the cementitious composite matrix is an effective method to increase material durability and reduce maintenance costs. Zone of inhibition studies of TiO_2_-infused cementitious samples showed efficacy toward both Gram-negative and Gram-positive bacteria.

## 1. Introduction

Worldwide, a shift in human lifestyles over the last half century has seen a growing number of daily activities move from the outdoors to enclosed inner spaces [1]. At the present time, it is known that the growth of micro-organisms (fungi, bacteria, viruses, algae, lichen, dust mites) on building surfaces (floors, walls, ceilings) has a detrimental effect on population health, particularly with contaminated interior surfaces. Maintaining good air quality and appearance of homes and workplaces has resulted in increased maintenance and repair costs. The existence of a so-called “Sick Building Syndrome (SBS)” manifests itself in the population that operates, partially or totally, inside buildings affected by mold or colonies (biofilms) of bacteria [1,2,3]. Pathogenic bacteria readily develop and survive on surfaces, especially in conditions of high humidity (min. 97%) and at temperatures ranging from −5 to +60 °C. The development of simple, inexpensive, and preventative antibacterial treatment methods of these surfaces are critical to maintain good public health [4,5]. In particular, areas of intensive medical use require the regular and thorough disinfection of surfaces in order to reduce the number of bacteria and prevent bacterial transmission to patients [4,5,6,7]. These untreated surfaces can act as reservoirs of microorganisms, which in turn could lead to the spread of infections [8]. Applications of the photocatalytic process of nano-TiO_2_ provides a conceptually simple and promising preventative technology for inhibiting contamination from bacteria, as well as an alternative to the constant use of chemical disinfectants [5,9,10,11].

The specific properties of TiO_2_ were discovered in the 1950s, but their exploitation in practical applications began only in 1972, when Fujishima and Honda used it for water splitting. Another very important property of TiO_2_ is its photoinduced superhydrophilicity, which was unexpectedly discovered in 1995 in SiO_2_/TiO_2_ composites illuminated by ultraviolet (UV) light.

An important feature of TiO_2_-SiO_2_ compounds is that, unlike TiO_2_, whose photocatalytic activity ceases without UV illumination, their photocatalytic effect continues for hours or even days after the removal of the UV source. In 1997, the first publication by Luigi Cassar et al. focused on the production of cementitious materials with self-cleaning properties [12]. A significant amount of research has shown the benefits of the introduction of TiO_2_ nanoparticles on the performance of cement composites: reduction of intake time, increase of the degree of cement hydration, increase of mechanical resistances (bending, compression, abrasion), adhesion to the reinforcement and resistance to its corrosion, and improvement of durability and freeze–thaw resistance [13,14,15,16].

In 1985, Matsunaga et al. first demonstrated the photocatalytic cytotoxic mechanisms in microbial cells from *Saccharomyces cerevisiae* (yeast), *Lactobacillus acidophilus* and *Escherichia coli* (bacteria), and *Chlorella vulgaris* (green algae) in water [17,18,19,20,21]. It is currently known that when the supplied photon energy is greater than the energy difference between the valence and conduction band edges of TiO_2_ (which occurs for UV radiation), photogenerated electrons (e−) and holes (h+) react with O_2_ and H_2_O, forming anionic radicals (O^2−^) and (OH). These oxidative species (h^+^, O^2−^, and OH) are highly reactive, contributing to the destruction of the cells of microorganisms [1,22,23,24,25,26]. The mechanism of destruction can be synthesized in the following sequence of reactions:

TiO_2_ + hν→TiO_2_ (e^−^*_cb_* + h^+^*_vb_*) (1)

O_2_ + e^−^*_cb_* → O^2−^ (2)

H_2_O + h^+^*_vb_* → •OH + H^+^ (3)

•OH + •OH → H_2_O_2_ (4)

O^2−^ + H_2_O_2_ → •OH + OH^−^ + O_2_ (5)

•OH + Organic + O_2_ → CO_2_ + H_2_O. (6)

A number of studies have been carried out in this direction. Sunada et al. [27] have shown that cell membranes are photocatalytically destroyed in the case of *Escherichia coli* bacteria. Saito et al. [28] proposed a mechanism for the destruction of bacteria by inhibiting their respiration function once they come into contact with TiO_2_. Oguma et al. [29] discussed a mechanism for the destruction of bacteria through the destruction of the cell wall and the induction of disorder at the cellular level following the contact of the microorganism with TiO_2_. Evidences highlight that wavelengths in the range of 320–400 nm are the most efficient to activate the photo-cytotoxic activity of TiO_2_. Gogniat et al. [30] showed that the adsorption capacity of TiO_2_ is positively correlated with its biocidal effect. Adsorption has been consistently associated with a reduction in the integrity of the bacterial membrane, as indicated by flow cytometry. The authors suggested that the adsorption of cells on photoactivated TiO_2_ is followed by a loss of membrane integrity, which was key to the biocidal effect. Mazurkova et al. [31] analyzed the effect of nano-TiO_2_ on the influenza virus, indicating the destruction of the virus in the presence of nanoparticles. After 15 min of incubation, the nanoparticles adhered to the outer surface of the virus, the surface spinules of the virus were glued together, and its outer membrane, of a lipoprotein nature, was torn. After 30 min, the degree of destruction increased, and after 1–5 h of incubation, the virus that came into contact with the nanoparticles was completely destroyed. It is considered that this effect depends on the duration of exposure/incubation, the viral concentration, and the concentration of nano-TiO_2_. The tests were carried out in three lighting conditions: dark, UV radiation, and natural light. Adams et al. [32] showed that the concentration of *Bacillus subtilis* and *Escherichia coli* were reduced upon contact with a suspension of nano-TiO_2_ under natural light illumination. A similar effect on *Bacillus subtilis* has been reported by Armelao et al. [33]. Research conducted by Dedkova et al. [34] on samples of kaolin composites containing nano-TiO_2_ indicated their biocidal effect in the presence of *Escherichia coli*, *Enterococcus faecalis*, and *Pseudomonas Aeruginosa*, after 2 days of exposure to artificial light. Results were also consistent with those of Gurr [35], who assessed that the antibacterial effect of TiO_2_ composites is manifested in the presence of natural light, without necessarily requiring UV photoactivation. The study carried out by Hamdani [36] has shown that based on the behavior of the two cementitious mixtures with 3% and 5% nano-TiO_2_-based type-Aeroxide P25, when in contact with *E. coli*, they were able to reduce the viability of bacteria after 24 h of exposure by 60–70%. The main observation, based on results in the literature [37,38,39], is that cementitious composite surfaces containing nano-TiO_2_ have the ability to inhibit biofilm growth, destroying the *E-coli bacteria* with which they come in contact. This is also supported by results obtained by Daly et al. [40], Carre et al. [41], and Kubacka et al. [42], which confirm that by the formation of free radicals and anions strongly oxidized by the photoactivation of nano-TiO_2_ (OH● and O_2_^−^), at the cellular level, plasma components such as DNA, RNA, lipids, and proteins are destroyed, and cell membranes are broken.

The easiest method of studying the resistance capacity of various building materials to the attack of microorganisms is the adapted antibiogram method, which is already used in medicine. This method is known as the halo inhibition method [43] or the Kirby–Bauer method, and it is currently standardized according to AATCC TM147 and AATCC TM30 (American Association of Textile Chemists and Colorists).

Although a large number of authors confirm the anti-bactericidal capacity of cement compounds with nano-TiO_2_, there is still controversy about maintaining the bactericidal capacity in the absence of light (during the night), as well as the influence of the type of bacteria that contaminate the surface. Results show that their destruction begins after only 20 min of UV radiation exposure and 60–120 min is sufficient to destroy all the bacteria [44], since hydroxyl radicals are the main factors responsible for the bactericidal capacity of semiconductor’s photocatalysts. They also possess a destruction capacity of *Escherichia coli* bacteria which is 10^3^–10^4^ times more effective than chemical disinfection products [45].

The aim of this work is to analyze the potential of inhibiting the growth of bacterial films on the surface of cementitious composites by adding different amounts of nano-TiO_2_, using the zone of inhibition to get a quick test of the antibacterial efficiency, and to identify the nanoparticle-functionalized regions in relation to the amount of cement, which ensure a successful effect of resistance from a biological point of view. For this purpose, four types of bacteria were used, which were chosen because of the frequency with which they are encountered in the building environment: *Escherichia coli*, *Pseudomonas Aeruginosa*, *Staphylococcus Aureus,* and *Streptococcus Pyogenes*.

## 2. Materials and Methods

In order to study the self-cleaning and anti-bacterial properties of cementitious composites, the literature shows three types of concrete modifications: concrete covered with a thin layer of TiO_2_, concrete covered by a thick layer of photoactive concrete on the top, and finally, different weight percentages of TiO_2_ in the concrete mass (when TiO_2_ substitutes cement or is present as additive) [46]. Each of these approaches has advantages and disadvantages in relation to the compatibility and adhesion to the substrate (in the case of thin films on the surface), durability, and impact on physical and mechanical performances (in the case of the introduction of TiO_2_ nanoparticles into the cementitious mass) and even economic impact in terms of costs [47]. The methodology used was based on the studies presented by Meija-De Gutierrez [43].

The preparation of the cementitious composites was performed by using white Portland cement as binder and by adding TiO_2_ nanoparticles as addition in different mass percentages, relative to cement quantity. Preliminary results obtained on the same mix-design ratios, when subjected to the method of staining with Rhodamine B and methylene blue, extended even for the situation of staining with exhaustion gas particles on the surface of the samples [48,49], have demonstrated the photocatalytic activity of nano-TiO_2_ addition in the cementitious matrix and thus the self-cleaning capacity of the samples. Furthermore, the influence of added TiO_2_ nanoparticles on the hydrophilicity of the cementitious composites surfaces was studied on the same mixtures [50].

Commercial Aeroxide P25 TiO_2_ nanoparticles, 99.5% purity (Evonik Industries AG, Hanau, Germany), containing more than 70% anatase, with a minor amount of rutile and a small amount of amorphous phase anatase and rutile crystallites, with a reported ratio of 70:30 or 80:20, were used in the production of the samples. Both phases play an important role in industrial applications and contribute to the photoactivation mechanism [3]. According to the manufacturer’s data sheet, the mean size of the TiO_2_ particles is 21 nm, with a specific surface of 35–65 m^2^/g. The test specimens were prepared and conditioned as shown in Table 1 in order to conduct the specific tests.

Then, the cementitious composite paste was cast into rectangular molds from which small, 17.4 mm circular samples were cut. Then, the samples were subjected to photoactivation, undergoing a 24 h UV ray treatment by using a light source with 400–315 nm spectrum emission (corresponding to the UVA band), which was located at a distance of 10 cm above the surface of the specimens, which determined a luminous flux intensity of 860 lux.

**Figure 1:**
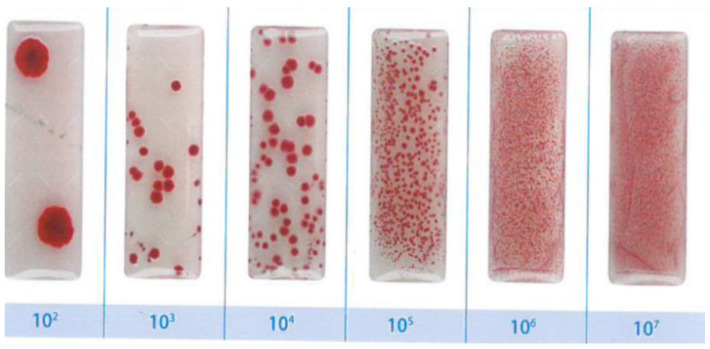
Quantification of biological load according to MicroKount^®^ specifications.

For testing resistance to the action of bacteria of the nano-TiO_2_ cementitious composite samples, *Escherichia coli* (ATCC 25922), *Pseudomonas Aeruginosa* (ATCC 27853), *Staphylococcus Aureus* (ATCC 25923), and *Streptococcus Pyogenes* (ATCC 19615) solutions have been used. These solutions were prepared by harvesting them from reference bacteria cultures and introducing 2 colonies (2 loops of 1 µL each) of biological material into 1 mL of physiological serum. The biological load of the prepared solutions was semi-quantified by the contact plate method, using the specifications of MicroKount^®^ microbilogic load test (Figure 1), determining a concentration of 10^7^ CFUs for each type of bacteria used.

At the same time, φ = 90 mm Petri dishes were prepared, in which suitable nutrient substrates were placed in order to develop the bacteria cultures, such as agar for bacteria cultures *Escherichia coli*, *Pseudomonas Aeruginosa*, and *Staphylococcus Aureus*, respectively, and blood agar for bacteria culture *Streptococcus Pyogenes*. All of them were sterilized under UV rays.

In each sterilized Petri dish with a suitable nutrient substrate, depending on the type of bacteria, 1 mL of bacterium suspension was applied and distributed so that the entire surface of the nutrient substrate was covered. Consequently, the photoactivated cementitious composite samples were centrally placed in the Petri dishes without any cross-contamination of the system.

Subsequently, 0.5 mL of biological material–bacterium suspension was applied on the samples, and the Petri dish lid was sealed by isolating the whole system on the edge to prevent cross contamination. One sample for each type of bacteria used was made without using any cementitious composite sample (P0). This was considered the primary control sample and was used to demonstrate the viability of the used bacteria in the suspensions.

Romanian STAS 12718 offers the possibility of semi-quantitative quantification of the microbiological load of the system, providing a quantification grid as follows: 0 (−) no growth (sterile); 1 (+) 1–10 colonies of microorganisms; 2 (++) over 10 colonies of microorganisms; 3 (+++) areas with confluent colonies; 4 (++++) growth throughout the surface.

Then, the farming systems were placed in the laboratory, at a temperature of 30 °C, under natural light conditions. As a result of exposure to natural light, the photoactivation process initially induced by UV exposure was continuously refreshed throughout 21 days, similarly to the alternation of day/night periods. Moreover, the initial UV exposure, for 24 h, ensured the sterilization of the cementitious samples so that there was no pre-existing contamination. At regular intervals of time, i.e., 2, 3, 4, 6, 7, 14, and 21 days, the development of the systems was examined visually and microscopically for signs of growth/development of the material (the colonies). The presence/development of the halo of inhibition was also studied. The quantification of the behavior of the bacteria contaminated samples was carried out qualitatively, according to STAS 12718 (Table 2) and using the Kirby–Bauer technique (AATCC TMII47), which is commonly used in medicine and has been known since 1966 as the antibiogram method. This method was adapted to the present requirements and conditions, by which the diameter of the inhibition halo, φ (mm) was measured.

The effectiveness of the antibacterial effect induced by nano-TiO_2_ photoactivation was assessed quantitatively by introducing a quantifiable parameter, EEA, which represents the percentage change in the diameter of the inhibition halo of the analyzed sample (with nano-TiO_2_ content in the matrix >0, relative to the control sample (with 0% nano-TiO_2_ content in the matrix), according to Equation (7).

EEA = (φ_%TiO2_ − φ_control_)/φ_control_ × 100 (%) (7)

where φ_%TiO2_—inhibition halo diameter measured for the sample with TiO_2_% > 0 (mm); φ_control_—inhibition halo diameter measured for TiO_2_ 0% control sample (mm).

## 3. Results and Discussion

**Figure 2:**
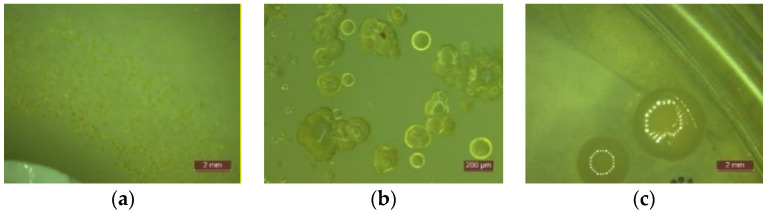
Microscopic examination of the behavior of samples exposed to *Escherichia coli*: (**a**) Identification of the inhibition halo area (1:2 mm); (**b**) Growth colonies (1:200 µm); (**c**) Detail of growth colonies (1:2 mm).

**Figure 3:**
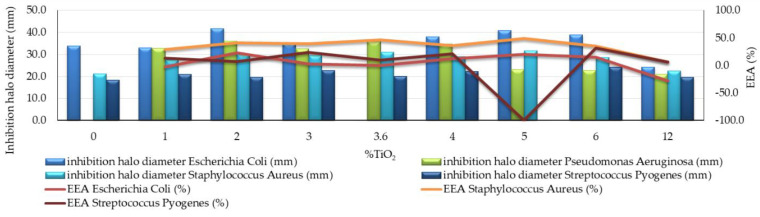
Inhibition halo size development after 2 days of exposure to bacteria *Escherichia coli*, *Pseudomonas Aeruginosa*, *Staphylococcus Aureus*, and *Streptococcus Pyogenes*. (In the case of *Pseudomonas Aeruginosa* contamination, the EEA could not be quantified, because the control sample (0% TiO_2_) did not show an inhibition halo). EEA: the percentage change in the diameter of the inhibition halo of the analyzed sample, indicating the effectiveness of the antibacterial effect.

**Figure 4:**
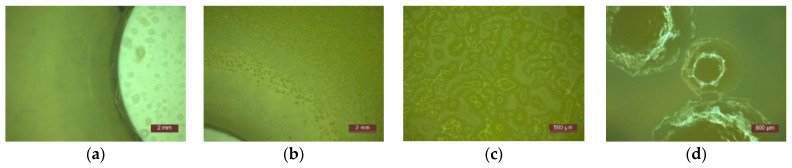
Microscopic examination of the behavior of samples exposed to *Pseudomonas Aeruginosa*: (**a**) The presence of biological material on the surface of the composite specimen at the time of sowing (1:2 mm); (**b**) Identification of the inhibition halo zone (1:2 mm); (**c**) Growth of colonies (1:500 µm); (**d**) Detail growth of colonies (1:500 µm).

**Figure 5:**
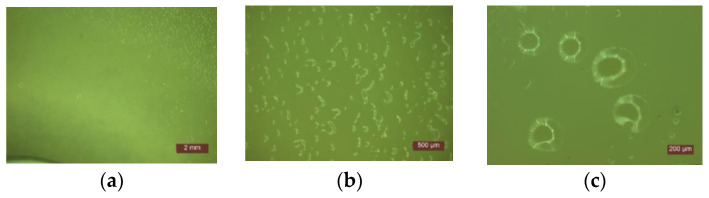
Microscopic examination of the behavior of samples exposed to *Staphylococcus Aureus*: (**a**) Identification of the inhibition halo area (1:2 mm); (**b**) Growth of colonies (1:500 µm); (**c**) Detail growth of colonies (1:200 µm).

**Figure 6:**
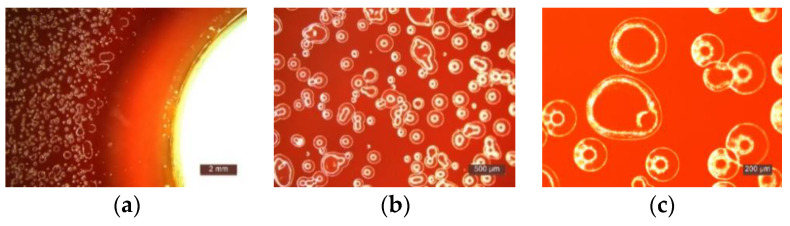
Microscopic examination of the behavior of samples exposed to *Streptococcus Pyogenes*: (**a**) Identification of the inhibition halo area (1:2 mm); (**b**) Growth of colonies (1:500 µm); (**c**) Detail growth of colonies (1:200 µm).

Experimental results on the behavior of cementitious composites with the addition of TiO_2_ in an environment contaminated with *Escherichia coli* are presented in Table 3 and in Figure 2 and Figure 3. For the environment contaminated with *Pseudomonas Aeruginosa*, results are presented in Table 4 and in Figure 3 and Figure 4. For the environment contaminated with *Staphylococcus Aureus*, results are shown in Table 5 and in Figure 3 and Figure 5 and for the samples contaminated with *Streptococcus Pyogenes*, results are shown in Table 6 and in Figure 3 and Figure 6.

**Figure 7:**
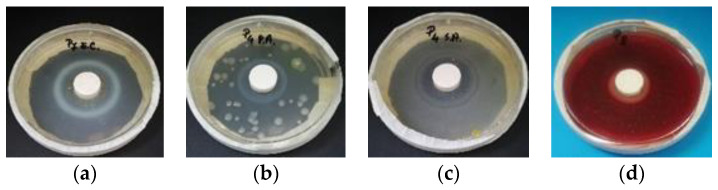
Nano-TiO_2_ systems evaluation: (**a**) 5% TiO_2_, (14 days exposure to *Escherichia coli*); (**b**) 3.6% TiO_2_ (6 days exposure to *Pseudomonas Aeruginosa)*; (**c**) 3% TiO_2_ (14 days exposure to *Staphylococcus Aureus*); (**d**) 6% TiO_2_ (21 days exposure to *Streptococcus Pyogenes*).

By analyzing the obtained results, several general features have been observed on all systems, regardless of the type of contaminant:

- No traces of contamination/development of bacterial colonies were observed on the surface of any of the tested cementitious material samples during the entire test period (21 days).
- In the first 48 h after exposure in the contaminated environment, the formation of inhibition haloes was observed, which remained constant in size and shape throughout the test. The systems presented a concentric shape: the cementitious composite sample being surrounded by a circular area with microbiological load, evaluated according to STAS 12718/1989, in Class 0 (-). This was followed by a zone of growth and the development of biological material. This increase was more intense as the distance from the edge of the cementitious composite increased (Figure 2, Figure 3, Figure 4, Figure 5, Figure 6 and Figure 7). The only exception was observed for samples tested with *Pseudomonas Aeruginosa*, for which no inhibition halo was identified in the cementitious composite control system.
- Sample P10 (12% TiO_2_) had in general a smaller halo diameter than samples with lower nanoparticle content. This behavior can be attributed to the inhomogeneity and the improper dispersion of nanoparticles in the cementitious matrix, which may tend to agglomerate.
- The P0 system, without the cementitious composite sample, had the most intense and rapid development of colonies of bacteria.
- Microscopic analysis revealed the presence and development of colonies of bacteria in the areas outside the inhibition halo, which again indicate the viability of the suspension used for seeding, the right choice of nutrient substrate, and exposure conditions.
- The formation of the inhibition halo for the control composite system (0% TiO_2_) also indicated resistance to the development of bacteria. This mainly happened because of the chemical composition of white Portland cement, which usually contains a certain quantity of TiO_2_.

In case of contamination with *Escherichia coli*, the following aspects could be identified:

- Cementitious composites with nano-TiO_2_ content in the range of 2–6% had the most effective behavior, with an efficiency of the antibacterial effect (EEA) of more than 10% (Figure 3). The highest value of this parameter (23%) was reported for the samples with 2% nano-TiO_2_ addition.
- When evaluating the entire system by quantifying the microbiological load of the system, according to STAS 12718 (Table 3), Classes 0 (-) or 1 (+) were observed/maintained for a longer period. It was also noticed that the samples with 4–12% nano-TiO_2_—(Class 0 (-))—maintain this sterile behavior longer (even after 7 days of exposure in contaminated environment—sample P6 (4% TiO_2_)). In addition, the P3 sample (2% TiO_2_) had a distinguished behavior, by keeping Class 1 (+) constant until the end of the test period.
- In the case of the P0 system, the formation of zones with confluent colonies (Class 3 (+++)) was observed earlier, after only 4 days at exposure in the contaminated environment. This confirmed the viability of the inoculated bacterial material.

In the case of contamination with *Pseudomonas Aeruginosa*, the following aspects can be identified:

- Due to the lack of visible and measurable inhibition halo in the control sample, the effectiveness of the antibacterial effect (EEA) could not be calculated, thus indicating a resistance effect to these bacteria of the cementitious composite matrix (Figure 3). However, for samples with 3.6% and 4% nano-TiO_2_, large inhibition halos have been observed.
- When evaluating the entire system by quantifying the microbiological load of the system, according to STAS 12718 (Table 4), Class 2 (++) was observed and maintained for a longer period. This happened due to the higher content of nanoparticles in the cementitious composite mass. For composite samples with 3.6–12% nano-TiO_2_, the framing Class 2 (++) was maintained throughout the 21 days of testing. This also happened for sample P4 (3% TiO_2_), whose framing class changed from 2 (++) to 3 (+++) only at the last stage of testing (during 14–21 days of exposure in the contaminated environment).
- In the case of the P0 system and the P1 control sample system (0% TiO_2_), the formation of areas with Class 3 (+++) confluent colonies was rapidly observed after 2 days of exposure in the contaminated environment, which on the one hand indicates the pre-viability of the bacterial inoculum material and on the other hand indicates the lack of antibacterial activity in the case of the P1 control composite matrix (0% TiO_2_) (Table 4).

In the case of contamination with *Staphylococcus Aureus*, the following aspects could be identified:

- Samples containing nano-TiO_2_ in the range of 1% to 5% had the most satisfactory behavior, i.e., an efficiency of the antibacterial effect (EEA) of more than 25% (Figure 3). The maximum effectiveness of the antibacterial effect (EEA) was achieved by the samples with 5% nano-TiO_2_, for which this indicator was 49%.
- The development of *Staphylococcus Aureus* colonies occurred less readily compared to the other types of bacteria analyzed in the study. The identified colonies were visible to the naked eye after only 2–3 days of exposure in the contaminated environment.
- In the case of the P0 system, colony formation Class 1 (+) was observed after 3 days of exposure, which confirms the viability of the inoculated bacterial material (Table 5).

In the case of contamination with *Streptococcus Pyogenes*, the following aspects could be identified:

- Samples with nano-TiO_2_ content in the range of 3–6% showed better behavior in terms of the ability to inhibit colony growth.
- When evaluating the entire system by quantifying the microbiological load of the system, according to STAS 12718 (Table 6), Classes 1 (+) or 2 (++) were observed and maintained during the first 2–3 days after exposure to the contaminated environment. For samples with 2–6% nano-TiO_2_, this classification was kept constant for up to 3 days. In these cases, the EEA quantifiable parameter reached the maximum value, i.e., 31%, for the 6% nano-TiO_2_ composition (Figure 3);
- In the case of the P0 system, the formation of more than 10 colonies, Class 2 (++) was observed after 2 days, Class 3 (+++) confluent colonies were observed after 3 days, and also Class 4 (++++) growth was observed throughout the surface (Table 6). Almost the same behavior, differentiated only by the delay in their development, was observed for P1 (0% TiO_2_), P2 (1% TiO_2_), and even the system with the maximum nanoparticle content, P10 (12% TiO_2_).

## 4. Conclusions

From the research performed, we can draw the following conclusions:

- The viability of the contaminants, selection of nutrients, and temperature conditions were proven. Therefore, the identification, quantification, and comparison between their action and the results regarding the growth of the biological material, when subjected to the cementitious composites, was demonstrated, based on the retention time of the samples in the contaminated environment and the content of nano-TiO_2_ in the samples.
- The effect of the development of the inhibition halo, when subjected to *Escherichia coli*, *Pseudomonas Aeruginosa*, and *Staphylococus Aureus* bacteria, namely, *Streptococcus Pyogenes* has also been confirmed for samples containing nano-TiO_2_ in the range of 2% to 5%. However, the introduction of large quantities of nanoparticles in the matrix of the composite may be on one hand beneficial in terms of antibacterial effects but, on the other hand, it is harmful as a result of the tendency of agglomeration of the nanoparticles in the matrix of the composite. Therefore, the effect of the antibacterial agent is considerably reduced.
- It was considered that for a good inhibiting activity against the development of contaminants of type *Escherichia coli*, *Pseudomonas Aeruginosa*, *Staphylococcus Aureus,* and *Streptococcus Pyogenes*, the content of TiO_2_ nanoparticles in the cementitious composite matrix should be at least 2% and not more than 5% relative to the amount of cement. The possibility remains open that composite samples with more than 5% nano-TiO_2_ are antibacterial effective if adequate nanoparticle dispersion is ensured. This range of identified nano-TiO_2_ amount is consistent with reports in the literature [36,37,38,39,40,41,42,51,52,53,54,55,56].

The results and observations presented in this paper can be a starting point for further research, consisting of a relatively fast and inexpensive semi-quantitative method for evaluating the antibacterial performance of cementitious composites with nano-TiO_2_. Through the work process adopted, it was also possible to highlight the antibacterial efficiency of cementitious composites with different nano-TiO_2_ percentages and to open new perspectives in the development of self-cleaning construction materials.

## Figures and Tables

**Table 1:** Mix ratio and conditioning of cementitious composites.

Mixture Number	P1	P2	P3	P4	P5	P6	P7	P8	P9	P10
Amount of nanoparticles relative to the amount of cement (%)	0	1	2	3	3.6	4	5	6	10	12
CEM I 52,5R white cement, HOLCIM (g)	500	500	500	500	500	500	500	500	500	500
Amount of water relative to the amount of total dry mixture (water/(cement + nano-TiO_2_)) (g)	0.5									
Conditioning	- 24 h in molds, 90% RH, 20 °C, without light; - demolding; - 27 days complete water immersion, 20 °C, without light.									

**Table 2:** Quantification of the biological load according to STAS 12718.

0 (-)	no growth (sterile)
1 (+)	1–10 colonies of microorganisms
2 (++)	over 10 colonies of microorganisms
3 (+++)	areas with confluent colonies
4 (++++)	growth throughout the surface

**Table 3:** Quantification of the microbiological load of the system according to STAS 12718 for samples exposed to *Escherichia coli*.

Exposure Period (Days)	P0 (without Composite Sample)	P1 (0% TiO_2_)	P2 (1% TiO_2_)	P3 (2% TiO_2_)	P4 (3% TiO_2_)	P5 (3.6% TiO_2_)	P6 (4% TiO_2_)	P7 (5% TiO_2_)	P8 (6% TiO_2_)	P10 (12% TiO_2_)
2	1 (+)	1 (+)	1 (+)	1 (+)	1 (+)	1 (+)	0 (-)	0 (-)	0 (-)	0 (-)
3	1 (+)	1 (+)	1 (+)	1 (+)	1 (+)	1 (+)	0 (-)	0 (-)	0 (-)	0 (-)
4	3 (+++)	1 (+)	1 (+)	1 (+)	1 (+)	1 (+)	0 (-)	0 (-)	0 (-)	0 (-)
6	3 (+++)	1 (+)	1 (+)	1 (+)	1 (+)	1 (+)	0 (-)	0 (-)	1 (+)	1 (+)
7	3 (+++)	1 (+)	1 (+)	1 (+)	1 (+)	1 (+)	0 (-)	1 (+)	1 (+)	1 (+)
14	3 (+++)	1 (+)	1 (+)	1 (+)	1 (+)	1 (+)	1 (+)	1 (+)	1 (+)	1 (+)
21	3 (+++)	1 (+)	3 (+++)	1 (+)	3 (+++)	3 (+++)	2 (++)	1 (+)	1 (+)	1 (+)

**Table 4:** Quantification of the microbiological load of the system according to STAS 12718 for samples exposed to *Pseudomonas Aeruginosa*.

Exposure Period (Days)	P0 (without Composite Sample)	P1 (0% TiO_2_)	P2 (1% TiO_2_)	P3 (2% TiO_2_)	P4 (3% TiO_2_)	P5 (3.6% TiO_2_)	P6 (4% TiO_2_)	P7 (5% TiO_2_)	P8 (6% TiO_2_)	P10 (12% TiO_2_)
2	3 (+++)	3 (+++)	2 (++)	2 (++)	2 (++)	2 (++)	2 (++)	2 (++)	2 (++)	2 (++)
3	3 (+++)	3 (+++)	2 (++)	2 (++)	2 (++)	2 (++)	2 (++)	2 (++)	2 (++)	2 (++)
4	3 (+++)	3 (+++)	2 (++)	2 (++)	2 (++)	2 (++)	2 (++)	2 (++)	2 (++)	2 (++)
6	3 (+++)	3 (+++)	2 (++)	2 (++)	2 (++)	2 (++)	2 (++)	2 (++)	2 (++)	2 (++)
7	3 (+++)	3 (+++)	3 (+++)	2 (++)	2 (++)	2 (++)	2 (++)	2 (++)	2 (++)	2 (++)
14	3 (+++)	3 (+++)	3 (+++)	3 (+++)	2 (++)	2 (++)	2 (++)	2 (++)	2 (++)	2 (++)
21	3 (+++)	3 (+++)	3 (+++)	3 (+++)	3 (+++)	2 (++)	2 (++)	2 (++)	2 (++)	2 (++)

**Table 5:** Quantification of the microbiological load of the system according to STAS 12718 for samples exposed to *Staphylococcus Aureus*.

Exposure Period (Days)	P0 (without Composite Sample)	P1 (0% TiO_2_)	P2 (1% TiO_2_)	P3 (2% TiO_2_)	P4 (3% TiO_2_)	P5 (3.6% TiO_2_)	P6 (4% TiO_2_)	P7 (5% TiO_2_)	P8 (6% TiO_2_)	P10 (12% TiO_2_)
2	0 (-)	0 (-)	0 (-)	0 (-)	0 (-)	0 (-)	0 (-)	0 (-)	0 (-)	0 (-)
3	1 (+)	1 (+)	0 (-)	0 (-)	0 (-)	0 (-)	0 (-)	1 (+)	0 (-)	0 (-)
4	1 (+)	1 (+)	0 (-)	0 (-)	1 (+)	0 (-)	0 (-)	1 (+)	0 (-)	1 (+)
6	1 (+)	1 (+)	1 (+)	0 (-)	1 (+)	0 (-)	1 (+)	1 (+)	0 (-)	1 (+)
7	1 (+)	1 (+)	1 (+)	0 (-)	1 (+)	1 (+)	1 (+)	3 (+++)	0 (-)	1 (+)
14	1 (+)	1 (+)	1 (+)	0 (-)	1 (+)	1 (+)	1 (+)	3 (+++)	1 (+)	1 (+)
21	1 (+)	1 (+)	1 (+)	1 (+)	1 (+)	1 (+)	1 (+)	3 (+++)	1 (+)	1 (+)

**Table 6:** Quantification of the microbiological load of the system according to STAS 12718 for samples exposed to *Streptococcus Pyogenes*.

Exposure Period (Days)	P0 (without Composite Sample)	P1 (0% TiO_2_)	P2 (1% TiO_2_)	P3 (2% TiO_2_)	P4 (3% TiO_2_)	P5 (3.6% TiO_2_)	P6 (4% TiO_2_)	P7 (5% TiO_2_)	P8 (6% TiO_2_)	P10 (12% TiO_2_)
2	2 (++)	2 (++)	1 (+)	1 (+)	1 (+)	1 (+)	1 (+)	1 (+)	1 (+)	2 (++)
3	3 (+++)	2 (++)	2 (++)	1 (+)	1 (+)	1 (+)	1 (+)	1 (+)	1 (+)	2 (++)
4	3 (+++)	2 (++)	2 (++)	2 (++)	2 (++)	2 (++)	2 (++)	2 (++)	2 (++)	2 (++)
6	3 (+++)	3 (+++)	3 (+++)	2 (++)	2 (++)	2 (++)	2 (++)	2 (++)	3 (+++)	3 (+++)
7	3 (+++)	3 (+++)	3 (+++)	2 (++)	2 (++)	2 (++)	2 (++)	3 (+++)	3 (+++)	3 (+++)
14	4 (++++)	4 (++++)	3 (+++)	3 (+++)	3 (+++)	3 (+++)	3 (+++)	3 (+++)	4 (++++)	4 (++++)
21	4 (++++)	4 (++++)	4 (++++)	3 (+++)	3 (+++)	3 (+++)	3 (+++)	3 (+++)	4 (++++)	4 (++++)

## Data Availability

The data presented in this study are available on request from the corresponding authors.
